# *Synechococcus* sp. PCC 7002 Performs Anoxygenic Photosynthesis and Deploys Divergent Strategies to Cope with H_2_S_n_ and H_2_O_2_

**DOI:** 10.3390/antiox14091122

**Published:** 2025-09-16

**Authors:** Yafei Wang, Yue Meng, Hongwei Ren, Ranran Huang, Jihua Liu, Daixi Liu

**Affiliations:** 1 School of Pharmaceutical Sciences, Cheeloo College of Medicine, Shandong University, Jinan 250012, China; 202521383@mail.sdu.edu.cn (Y.W.); 202116948@mail.sdu.edu.cn (Y.M.); 2 Institute of Marine Science and Technology, Shandong University, Qingdao 266237, China; huangrr@sdu.edu.cn (R.H.); liujihua1982@sdu.edu.cn (J.L.); 3 Key Laboratory of Land and Sea Ecological Governance and Systematic Regulation, Ministry of Ecology and Environment, Shandong Academy for Environmental Planning, Jinan 250101, China; renhongwei@shandong.cn

**Keywords:** anoxygenic photosynthesis, oxygenic photosynthesis, reactive sulfur species, reactive oxygen species, *Synechococcus* sp. PCC 7002, transcriptome

## Abstract

Oxygenic and anoxygenic photosynthesis have long been considered defining traits of cyanobacteria. However, whether the important cyanobacterial genus *Synechococcus* is capable of anoxygenic photosynthesis remains unconfirmed. Here, we report that *Synechococcus* sp. PCC 7002 is capable of anoxygenic photosynthesis when sulfide (H_2_S) is supplied as the sole electron donor. Combining the targeted deletion of the sulfide: quinone oxidoreductase gene (Δ*sqr*) with 3-(3,4-dichlorophenyl)-1,1-dimethylurea (DCMU) mediated the inhibition of photosystem II. We demonstrated that SQR-mediated H_2_S oxidation sustains light-dependent CO_2_ fixation in the absence of O_2_ evolution. Our genome-wide transcriptomic profiling further revealed that polysulfide (H_2_S_n_) and hydrogen peroxide (H_2_O_2_) function as distinct signaling molecules in oxygenic and anoxygenic photosynthesis, modulating central carbon and energy metabolism. In central carbon metabolism, H_2_S_n_ markedly upregulates the expression of key genes, including *psbA*, *petC*, *rbcL*, and *rbcS*, whereas H_2_O_2_ downregulates these genes. Within energy metabolism, both molecules converge on oxidative phosphorylation by upregulating genes encoding NADH dehydrogenase and ATP synthase. Furthermore, H_2_Sₙ treatment uniquely induces sulfur-assimilation and ROS-detoxifying enzymes, conferring a markedly higher tolerance than H_2_O_2_. These findings provide direct evidence of anoxygenic photosynthesis in the genus *Synechococcus* and uncover a dual regulatory network that allows *Synechococcus* sp. PCC 7002 to balance redox homeostasis under fluctuating oxic/anoxic conditions.

## 1. Introduction

Cyanobacteria are an ancient group of prokaryotes capable of both oxygenic and anoxygenic photosynthesis, thereby occupying a pivotal position in the global carbon, nitrogen, and sulfur cycles [1,2]. As the earliest organisms to evolve oxygenic photosynthesis, cyanobacteria split water during this process and release molecular oxygen into the atmosphere [3,4,5], driving the transition of Earth’s atmosphere from a reducing to an oxidizing state and creating the preconditions for the subsequent evolution of aerobic life. Anoxygenic photosynthesis proceeds without O_2_ evolution and relies on alternative electron donors [6]. Certain cyanobacteria can use H_2_S as the electron donor in anoxygenic photosynthesis [7], thereby fixing CO_2_ into organic matter. This metabolic flexibility enables survival under extreme environmental settings. During the early days of the Earth, when the atmospheric O_2_ concentrations were extremely low, anoxygenic photosynthesis enabled cyanobacteria to thrive in primordial, hostile habitats, laying the foundation for the later evolution of oxygenic photosynthesis. *Synechococcus* is a prominent genus within the cyanobacteria, renowned for its global distribution and physiological diversity [8]. Although generally considered obligate oxygenic phototrophs, some evidence suggests that, under particular environmental conditions, certain *Synechococcus* strains may possess the potential to perform anoxygenic photosynthesis. For example, in habitats with elevated sulfide concentrations, *Synechococcus* might exploit H_2_S as an electron donor to alleviate oxidative stress and maintain cellular homeostasis [9]; however, definitive experimental proof is still lacking. Verifying of anoxygenic photosynthetic capacity in *Synechococcus* would provide a more comprehensive understanding of its metabolic versatility under fluctuating environmental conditions, illuminate the adaptive mechanisms that enabled the shift from anoxic to oxic environments across cyanobacterial evolution, and furnish crucial clues for reconstructing the history of early life on Earth.

The fundamental difference between oxygenic and anoxygenic photosynthesis lies in the choice of electron donor and the concomitant production (or absence) of molecular oxygen. Reactive oxygen species (ROS) and reactive sulfur species (RSS) are intermediates generated during these two modes of photosynthesis and function as signaling molecules that modulate cellular processes. Oxygenic photosynthesis produces ROS such as hydrogen peroxide (H_2_O_2_), superoxide anion (O_2_^−^), and hydroxyl radical (OH·). These ROS act as oxidative stress signals that coordinate cellular responses to environmental fluctuations [10]. When cyanobacteria are subjected to lots of light, nutrient limitation, or heavy metal stress, ROS levels increase, thereby activating a suite of protective mechanisms. ROS further modulate the activity of multiple transcription factors, leading to the altered expression of antioxidant enzymes and metabolic genes [11]. In contrast, anoxygenic photosynthesis yields RSS, including hydrogen persulfide (H_2_S_2_) and its derivatives (e.g., polysulfides, H_2_S_n_) [12]. RSS are intimately linked to sulfur metabolism and sulfur-dependent signaling. They participate in the regulation of intracellular sulfur homeostasis by influencing the uptake, transformation, and utilization of sulfur. RSS can react with cysteine residues of proteins to generate persulfidation modifications, thereby altering protein activity and function [13]. Although high concentrations of RSS are potentially toxic, at low levels they may serve as antioxidants that protect cells from oxidative damage [14]. Collectively, ROS and RSS fulfill distinct signaling roles via separate pathways and influence divergent physiological processes. Nevertheless, for cyanobacteria capable of both photosynthetic modes, a systematic, genome-wide investigation of ROS- and RSS- mediated signaling has not been performed.

Although ROS and RSS are indispensable for signal transduction, supraphysiological concentrations impose oxidative or sulfide stress, and organisms have evolved multifaceted detoxification strategies. Bacteria eliminate ROS via enzymatic antioxidants such as superoxide dismutase (SOD), catalase (CAT), and glutathione peroxidase (GPx) [15,16,17]. SOD converts O_2_^−^ to H_2_O_2_, which is subsequently decomposed to H_2_O and O_2_ via CAT and GPx, thereby alleviating ROS toxicity. Low-molecular-weight antioxidants, including glutathione (GSH) and ascorbate (AsA), directly scavenge ROS by donating electrons or hydrogen atoms, thus preventing oxidative damage. Sulfide detoxification is achieved through sulfide: Quinone oxidoreductase (SQR) [18] and flavocytochrome c sulfide dehydrogenase (FCSD), which oxidize H_2_S to polysulfides. Also, polysulfides are further converted to harmless thiosulfate via persulfide dioxygenase (PDO) [19,20,21,22,23,24]. Despite the distinct metabolic pathways for ROS and RSS, commonalities exist in bacterial defense systems. For instance, the ROS-detoxifying enzyme peroxiredoxin (Prx) can also reduce sulfane sulfur to H_2_S, and the ROS-sensing transcriptional regulators OxyR and PerR are capable of detecting RSS and modulating their metabolism [25,26]. Because cyanobacteria can switch between oxygenic and anoxygenic photosynthesis, they must coordinate ROS and RSS metabolizing pathways to sustain redox homeostasis under variable environmental conditions. A systematic, genome-wide elucidation of these pathways in cyanobacteria is therefore essential to understand their environmental adaptability, yet such analyses remain scarce.

In this study, we experimentally demonstrated that *Synechococcus* sp. (Cyanobacteria) PCC 7002 can perform anoxygenic photosynthesis via the SQR-mediated oxidation of H_2_S by deleting the *sqr* gene and pharmacologically inhibiting oxygenic photosynthesis with DCMU. Subsequent transcriptomic profiling was utilized to dissect, on a genome-wide scale, the regulatory effects exerted by H_2_O_2_ and H_2_S_n_ as signaling molecules on key genes involved in photosynthesis, the tricarboxylic acid (TCA) cycle, glycolysis, and oxidative phosphorylation. The data reveal extensive overlap in the signaling functions of H_2_O_2_ and H_2_S_n_, but the cells display heightened sensitivity to RSS. Moreover, *Synechococcus* sp. PCC 7002 exhibits superior tolerance to H_2_S_n_, which not only induces the expression of sulfur-metabolizing enzymes but also activates antioxidant systems. Collectively, our findings delineate a differential response strategy employed by *Synechococcus* to ROS and RSS, providing a theoretical framework for dissecting the regulatory mechanisms underlying distinct photosynthetic modes and the adaptation of cyanobacteria to the transition from anoxic to oxic environments.

## 2. Materials and Methods

### 2.1. Strains and Culture Conditions

*Synechococcus* sp. PCC 7002 was kindly provided by the Marine Microbial Ecology Laboratory, Institute of Marine Science and Technology, Shandong University, under a non-commercial material transfer agreement. *Synechococcus* sp. PCC 7002 and its mutant strain were cultured in conical flasks containing medium A [27], supplemented with 1mg of NaNO_3_ mL^−1^ (designed as medium A+), at 30 °C under continuous illumination with 50 μmol of photons m^−2^ s^−1^ on a shaker set at 150 rpm. Glycerol (10 mM) was added as a supplement in the medium A+ to serve as the carbon and energy source. The Δ*sqr* mutant of *Synechococcus* sp. PCC 7002 was constructed using a homologous recombination as described in a previous study [23]. For the mutant strain, the appropriate antibiotic, 50 µg mL^−1^ of kanamycin, was added.

### 2.2. Verification of Anoxygenic Photosynthesis

To verify whether the *Synechococcus* sp. PCC 7002 can utilize H_2_S as an electron donor for anoxygenic photosynthesis under the action of SQR, the following experiments were performed: DCMU at a concentration of 0.5 µM was employed to inhibit the oxygen-evolving photosynthetic process, thereby ensuring that the observed photosynthesis in the experiment was anoxygenic. Subsequently, H_2_S was introduced into the culture medium at concentrations of 250 µM and 500 µM, respectively, to examine the photosynthetic capabilities of *Synechococcus* sp. PCC 7002 under different H_2_S concentrations. To maximize the removal of oxygen from the culture medium, nitrogen gas was bubbled through it, establishing an anaerobic environment for the experiment. The treated culture medium was then placed in an anaerobic culture bottle for cultivation. After inoculation, both the wild-type *Synechococcus* sp. PCC 7002 and Δ*sqr* strains were cultured under the continuous illumination of 50 µmol of photons m^−2^ s^−1^ at 30 °C, with shaking at 150 rpm. During the cultivation process, cell growth was continuously monitored for 15 days, with cell densities measured spectrophotometrically at OD_730_ to track the growth status.

### 2.3. Induction Experiments with H_2_S_n_ and H_2_O_2_

To investigate the effects of H_2_S_n_ and H_2_O_2_ on cyanobacteria in the logarithmic growth phase, we designed the following induction experiments. First, *Synechococcus* sp. PCC7002 was cultured to the logarithmic growth phase. Subsequently, the concentration gradients of H_2_S_n_ and H_2_O_2_ were established at 250 µM, 500 µM, and 1000 µM, with three biological replicates for each concentration to ensure that the experimental results were reliable and could be reproduced. *Synechococcus* sp. PCC7002 was then exposed to H_2_S_n_ and H_2_O_2_ and subjected to induction treatment for 1 h under shaking conditions at 150 rpm and 30 °C. After the induction, cells were immediately collected via centrifugation (4000× *g*, 4 °C, 10 min) and washed twice with precooled physiological saline to remove residual inducers. Finally, the collected cells were rapidly frozen and stored at −80 °C to ensure that they can be used as high-quality samples for subsequent transcriptomic analysis.

### 2.4. RNA Extraction and Transcriptome Sequencing

Total RNA was isolated using the TaKaRa MiniBEST Universal RNA Extraction Kit (TaKaRa, Beijing, China), and the RNA concentration was quantified with a Qubit 4 instrument (Thermo Fisher, Shanghai, China). The RNA-seq libraries were constructed with the NEBNext^®^ Ultra II™ Directional RNA Library Prep Kit (New England Biolabs, Ipswich, MA, the US) for Illumina in accordance with the manufacturers’ instructions. Three independent biological replicates were included for each sample. The transcriptome was sequenced using the Illumina HiSeq 2500 platform (Illumina, San Diego, CA, USA) by Magigene.

### 2.5. RNA-Sequencing Analysis

For quality control purposes, we used fastp (v0.19.7) [28] to remove adapter sequences and low-quality reads from the raw reads to yield high-quality clean reads. Then, the clean reads were aligned to the Rfam ribosomal RNA (rRNA) database [29] using Bowtie2 (v2.33) [30], and the reads that were not mapped to the rRNA sequences were retained for use in the downstream analysis. The filtered reads were then aligned to the reference genome using HISAT2 (v2.1.0) [31]. Gene expression levels were quantified with RSEM (v1.2.12) [32] and normalized using the fragments per kilobase of transcript per million mapped reads (FPKM) method.

Subsequently, read count data were used as the input for differential gene expression analysis with the edgeR (v3.20.2) [33] package in R. Differentially expressed genes (DEGs) were identified using a negative binomial model for each comparison group. *p*-values were adjusted for multiple tests using the Benjamini–Hochberg (BH) [34] method to control the false discovery rate (FDR). Genes with FDR ≤ 0.05 and |log_2_ (fold change)| ≥ 1 were considered to be significantly differentially expressed. The overall distribution and significance of DEGs were visualized using volcano plots generated with the ggplot2 package in R. In addition, the heat map was drawn using chiplot (https://www.chiplot.online/, accessed on 5 June 2025).

Significantly differentially expressed genes were subjected to Gene Ontology (GO) [35,36] and Kyoto Encyclopedia of Genes and Genomes (KEGG) [37] enrichment analyses using the clusterProfiler [38] (v3.4.4) R (4.3.1) package. For KEGG pathway enrichment, *p*-values were corrected using the Benjamini–Hochberg (BH) [34] method, and pathways with an FDR ≤ 0.05 were considered to be significantly enriched among the candidate DEGs. The enrichment results were visualized using bubble plots to highlight the top enriched biological pathways. Additionally, operon structures and transcription start sites (TSSs) were analyzed using Rockhopper (v2.0.3) [39].

### 2.6. Tolerance of Synechococcus sp. PCC7002 to H_2_S_n_ and H_2_O_2_

To evaluate the tolerance of *Synechococcus* sp. PCC 7002 to H_2_S_n_ and H_2_O_2_, the following experiments were performed: Initially, *Synechococcus* sp. PCC 7002 cells in the logarithmic growth phase were harvested and treated with H_2_S_n_ and H_2_O_2_ at concentrations of 1 mM, 2 mM, 3 mM, 4 mM, and 5 mM for 6 h. During the treatment, the cells were cultured under consistent experimental conditions at 30 °C with shaking at 150 rpm. After the treatment, cells were collected using centrifugation (4000× *g*, 4 °C, 10 min) and washed twice with precooled physiological saline to remove residual H_2_S_n_ and H_2_O_2_. Subsequently, the treated *Synechococcus* sp. PCC 7002 cells were subjected to serial dilution with dilution factors of 10, 10^−1^, 10^−2^, 10^−3^, and 10^−4^. The diluted cells were then spotted onto solid medium A+, with three replicates for each dilution factor. Finally, the spotted culture dishes were incubated at 30 °C in a constant-temperature incubator for 7 days, and we recorded the changes in growth status were recorded.

### 2.7. Data Availability

Raw sequencing reads were deposited in the NCBI Sequence Read Archive (SRA) under project number PRJNA1289363.

## 3. Results

### 3.1. Synechococcus sp. PCC7002 Performed Anoxygenic Photosynthesis

**Figure 1:**
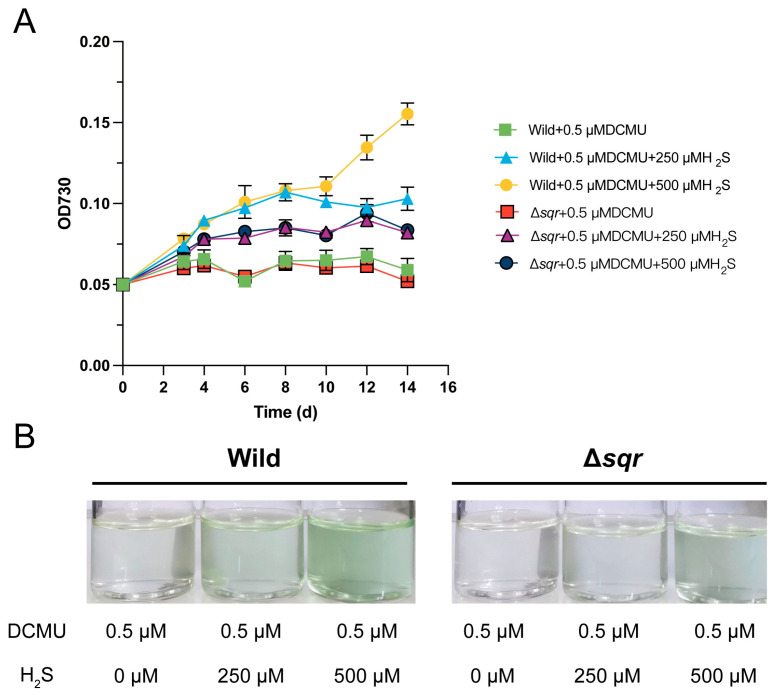
SQR-dependent anoxygenic growth of *Synechococcus* sp. PCC 7002. (**A**) Growth kinetics of *Synechococcus* sp. PCC 7002 (Wild) and the SQR-deficient mutant (Δ*sqr*) cultured under strict anaerobiosis in medium supplemented with 0.5 µM DCMU. Cultures were additionally supplied with 0, 250 or 500 µM H_2_S. Optical density at 730 nm (OD_730_) was monitored for 15 days. Data points represent the mean of three biological replicates; error bars indicate ± SD. (**B**) Representative photographs of the cultures after 15 days of incubation. *Synechococcus* sp. PCC 7002 cells (Wild) recovered normal growth in the presence of 250–500 µM H_2_S, whereas the SQR-deficient mutant (Δ*sqr*) remained arrested regardless of H_2_S addition, confirming that SQR-mediated H_2_S oxidation is essential for anoxygenic photosynthetic growth.

To determine whether *Synechococcus* sp. PCC7002 is capable of anoxygenic photosynthesis, we employed DCMU to selectively abolish oxygenic photosynthesis. DCMU specifically blocks electron transfer from the primary quinone acceptor (QA) to the secondary quinone acceptor (QB) in photosystem II (PSII). At 0.5 µM, DCMU completely arrested cell proliferation of *Synechococcus* sp. PCC7002; however, supplementation with H_2_S alleviated this inhibition (Figure 1 and Figure S1). After 10 days of anaerobic cultivation, the optical density of cultures containing 0.5 µM of DCMU alone remained extremely low. In contrast, cultures additionally supplied with 250 µM or 500 µM of H_2_S reached densities comparable to the untreated control (Figure 1). Partial recovery was observed at 1 mM of H_2_S. Still, no beneficial effect was seen at 2 mM, presumably because H_2_S becomes toxic to *Synechococcus* sp. PCC7002 at this concentration (Figure S1). In addition, SQR is indispensable for anoxygenic photosynthesis in *Synechococcus* sp. PCC 7002. To corroborate its functional role, we repeated the above assays using the SQR-deficient mutant we had previously constructed [23]. Earlier work has demonstrated that the deletion of SQR does not impair growth under normal photoautotrophic conditions; rather, it elevates the O_2_-evolution rate, the maximum photochemical efficiency of PSII (Fv/Fm), the relative electron transport rate (rETR), and the transcript levels of key photosynthetic genes, collectively indicating an enhanced oxygenic photosynthetic capacity. Here, when PSII was pharmacologically inhibited via DCMU, loss of SQR markedly suppressed growth and completely abolished the H_2_S-mediated rescue. These findings provide direct evidence that *Synechococcus* sp. PCC 7002 acquires electrons via the SQR-dependent oxidation of H_2_S to sustain anoxygenic photosynthesis.

### 3.2. The Transcriptional Response of Synechococcus sp. 7002 to H_2_S_n_ and H_2_O_2_

**Figure 2:**
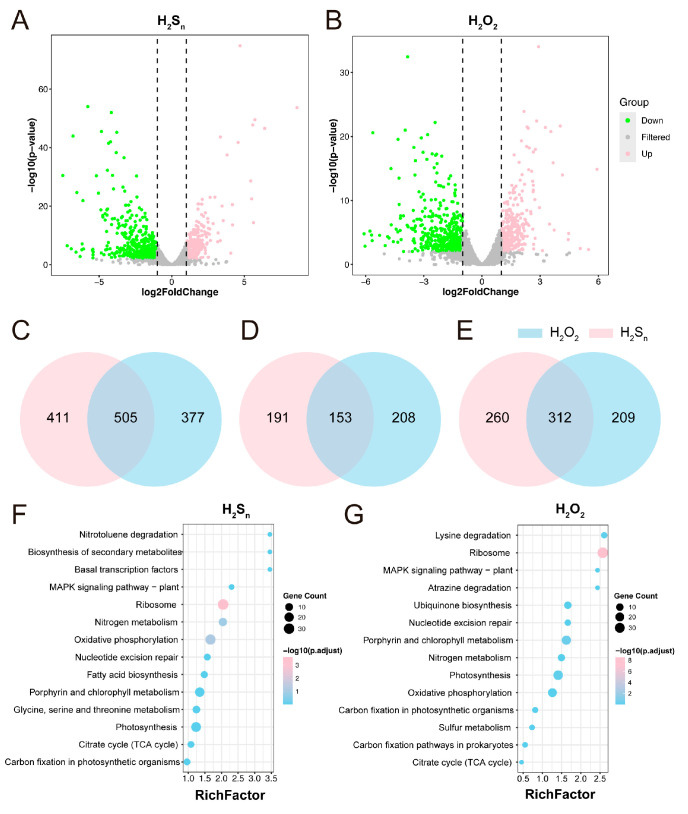
The transcriptional response of *Synechococcus* sp. PCC7002 to H_2_S_n_ and H_2_O_2_. (**A**,**B**) Volcano plots displaying differentially expressed genes (DEGs) after 1 mM H_2_S_n_ (**A**) or 1 mM H_2_O_2_ (**B**) treatment for 60 min. Horizontal dashed lines denote the adjusted *p*-value threshold (padj < 0.05); vertical dashed lines mark |log_2_ FC| ≥ 1. (**C**) Venn diagram comparing the total sets of DEGs (up- and downregulated) between the two treatments. (**D**,**E**) Overlaps among upregulated (**D**) and downregulated (**E**) genes in response to H_2_S_n_ and H_2_O_2_. (**F**,**G**) KEGG pathway enrichment analyses of DEGs following H_2_S_n_ (**F**) and H_2_O_2_ (**G**) exposure. Bubble size indicates the number of enriched genes; color intensity represents log_10_ (padj); and the *x*-axis (RichFactor) reflects the proportion of DEGs relative to all genes in each pathway. The dashed line indicates a log2FoldChange of 1.

To thoroughly investigate the effects of intermediate products from different photosynthetic modes on the key metabolic processes of *Synechococcus* sp. PCC7002, we selected H_2_S_n_ produced via anoxygenic photosynthesis and H_2_O_2_ produced via oxygenic photosynthesis as the subjects of this investigation. We treated *Synechococcus* sp. PCC7002 cells in the logarithmic growth phase with H_2_S_n_ and H_2_O_2_ at concentrations of 250 µM, 500 µM, and 1000 µM for 1 h. Each concentration was replicated three times to ensure the reliability of the experimental results. After treatment, cell samples were collected for transcriptomic analysis. Using high-throughput sequencing technology, we obtained a total of 42 GB of raw data (Figure S2). PCoA of log-FPKM profiles (Bray–Curtis) separated samples along PCo1 (33.54% variance) by oxidant identity (H_2_O_2_ vs. H_2_S_n_), with minor concentration-dependent shifts along PCo2, revealing distinct and dose-responsive transcriptomic signatures (Figure S3). Volcano plot analysis was performed and the results revealed that after treatment with 1000 µM of H_2_S_n_, a total of 916 genes in *Synechococcus* sp. PCC7002 cells were differentially expressed, with 344 upregulated (log_2_FC > 1) and 572 downregulated genes (log_2_FC < −1) (Figure 2A and Figure S4). In contrast, after treatment with 1000 µM of H_2_O_2_, the total number of differentially expressed genes was 882, with 361 upregulated genes (log_2_FC > 1) and 521 downregulated genes (log_2_FC < −1) (Figure 2B). Further analysis of all differentially expressed genes (DEGs) showed that 505 genes exhibited the same expression changes after treatment with both H_2_S_n_ and H_2_O_2_, with 153 upregulated and 312 downregulated genes being identical. Venn diagram analysis revealed that there were 505 differentially expressed genes (DEGs) shared between the H_2_S_n_ and H_2_O_2_ treatments, of which 153 were co-upregulated and 312 were co-downregulated (Figure 2C–E). To further explore the functions of these DEGs in cellular metabolic processes, we performed KEGG enrichment analysis on all DEGs (Figure 2F,G and Figure S5). The results indicated that the DEGs after treatment with 1000 µM of H_2_S_n_ and H_2_O_2_ were mainly enriched in key metabolic pathways such as photosynthesis, oxidative phosphorylation, carbon fixation, and chlorophyll synthesis. This finding suggests that despite originating from different photosynthetic modes, H_2_S_n_ and H_2_O_2_ exhibit certain similarities in their regulatory effects on the key metabolic processes of *Synechococcus* sp. PCC7002. In summary, as important signaling molecules from anoxygenic and oxygenic photosynthesis, H_2_S_n_ and H_2_O_2_ have both similarities and differences in their effects on the transcriptional level of *Synechococcus* sp. PCC7002. This study provides important theoretical insights into the mechanisms by which photosynthetic intermediates regulate the metabolism of Cyanobacteria.

### 3.3. The Effect of H_2_S_n_ and H_2_O_2_ on Photosynthesis of Synechococcus sp. PCC7002

**Figure 3:**
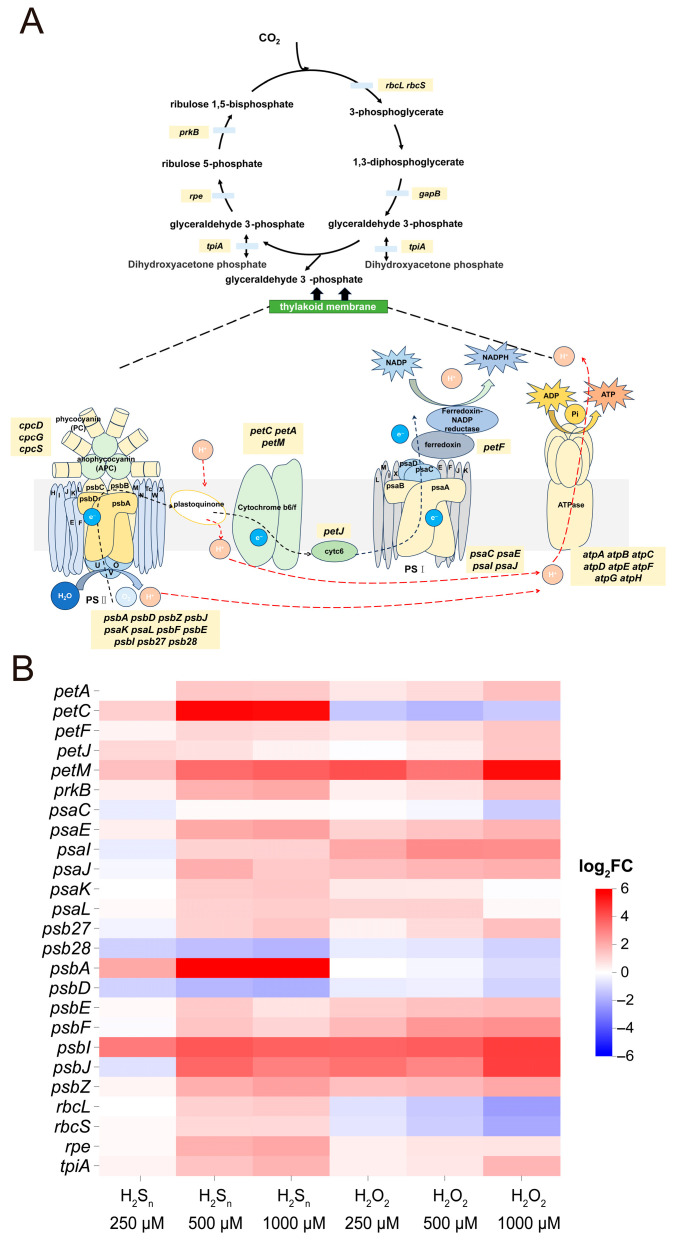
The effect of H_2_S_n_ and H_2_O_2_ on photosynthesis process of *Synechococcus* sp. PCC7002. (**A**) Schematic of the Calvin–Benson–Bassham cycle and the photosynthetic electron-transport chain (thylakoid membrane) highlighting genes that were differentially expressed (|log_2_ FC| ≥ 1, padj < 0.05) after exposure to 250, 500 or 1000 µM H_2_S_n_ and H_2_O_2_. The details of Figure 3 were adapted from the work of Dr. Donald A. Bryant, Dr. Jindong Zhao, and colleagues [40,41]. (**B**) Heat map displaying the log_2_ fold-change values of all photosynthesis-related DEGs.

We explored the mechanisms by which H_2_S_n_ and H_2_O_2_ affect photosynthesis, with a particular focus on light reactions and the Calvin cycle (Figure 3A). The results indicated that the effects of H_2_S_n_ and H_2_O_2_ treatments on photosynthesis generally followed a similar trend: the majority of DEGs were primarily concentrated in photosystem II (PSII), ATPase, and the Calvin cycle, with partial changes also observed in the encoding genes of the cytochrome b6f complex and photosystem I (PSI). The pattern of differentially expressed genes was such that in the presence of H_2_S_n_ and H_2_O_2_, the expression of most genes was significantly upregulated, and as the concentration increased, the fold change in upregulation also correspondingly increased (Figure 3B). In addition to upregulated genes, some genes exhibited downregulation, such as *atpE*, *atpF*, *psbA*, *atpC*, *psb28*, *psbD,* and *atpG*. We paid particular attention to genes that showed opposite patterns of change after H_2_S_n_ and H_2_O_2_ treatments. For instance, the *psbA* gene was significantly upregulated after H_2_S_n_ treatment, with fold changes of 2.00, 8.31, and 8.63, respectively, while it was downregulated after H_2_O_2_ treatment. The *petC* gene was upregulated by 1.17, 5.64, and 5.58 folds after H_2_S_n_ treatment but downregulated by 1.35, 1.74, and 1.30 folds after H_2_O_2_ treatment, respectively. Additionally, the *rbcL* and *rbcS* genes, encoding the large and small subunits of Rubisco, respectively, were upregulated after H_2_S_n_ treatment but downregulated after H_2_O_2_ treatment. These findings suggest that although H_2_S_n_ and H_2_O_2_ share similarities in the regulation of the expression of some photosynthesis-related genes, they may influence photosynthesis through different signaling pathways, especially in terms of the transcriptional regulation of PSII repair (*psbA*), the electron transport chain (*petC*), and the key enzyme of carbon fixation (Rubisco). This difference may reflect the distinct regulatory mechanisms of the two signaling molecules in photosynthesis, providing an important molecular basis for a deeper understanding of the fine regulation of photosynthesis.

### 3.4. The Effect of H_2_S_n_ and H_2_O_2_ on TCA Cycle, Glycolysis, and Oxidative Phosphorylation of Synechococcus sp. PCC7002

**Figure 4:**
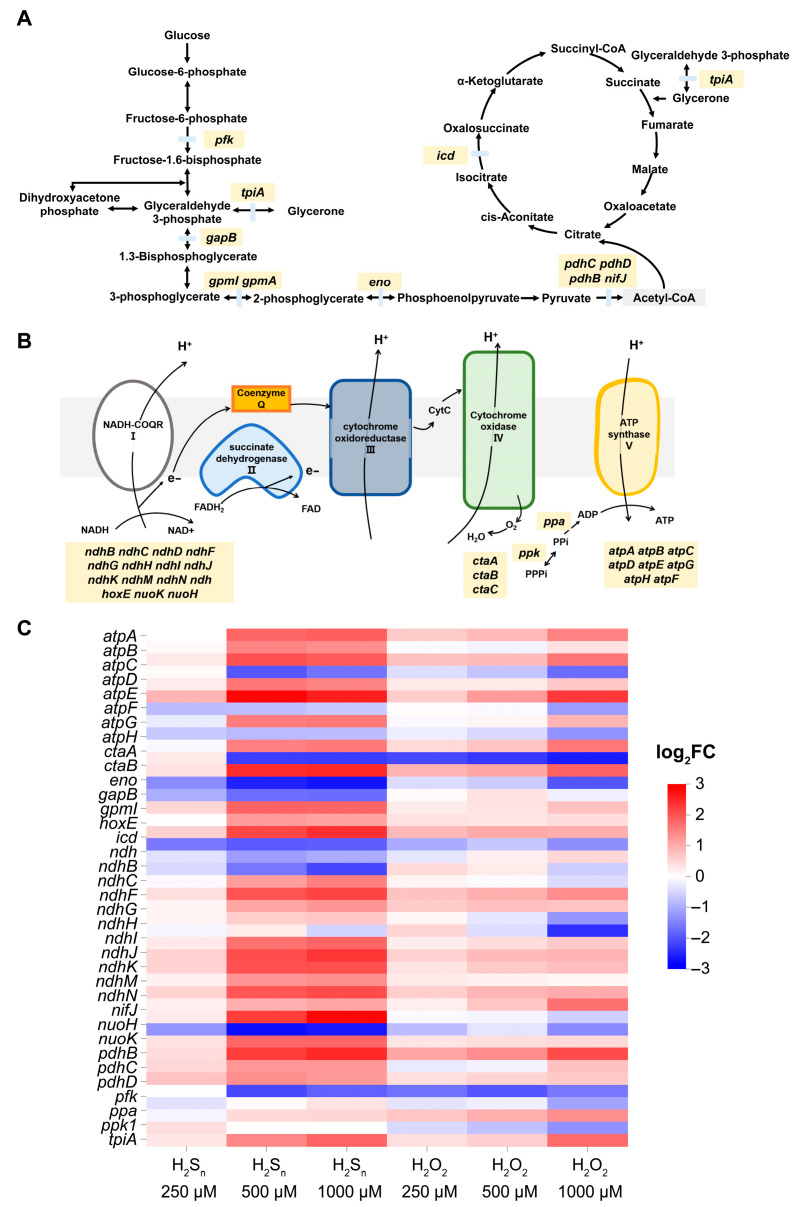
The effect of H_2_S_n_ and H_2_O_2_ on central carbon metabolism and energy production of *Synechococcus* sp. PCC7002. (**A**) Schematic overview of the TCA cycle and glycolysis indicating differentially expressed genes (|log_2_ FC| ≥ 1, padj < 0.05) after exposure to 250, 500 or 1000 µM H_2_S_n_ and H_2_O_2_. (**B**) Simplified oxidative-phosphorylation electron-transport chain highlighting the same DEG sets. Complexes I–V are shown with their corresponding gene products. (**C**) Heat map of log_2_ fold-changes for all DEGs associated with glycolysis, the TCA cycle and oxidative phosphorylation. The color scale ranges from −3 (strong repression, blue) to +3 (strong induction, red).

Furthermore, we explored the potential regulatory mechanisms of H_2_S_n_ and H_2_O_2_ on the key aspects of cellular energy metabolism, including the TCA cycle, glycolysis (Figure 4A), and oxidative phosphorylation (Figure 4B). The results revealed that a total of 42 genes exhibited significant changes in their expression levels under the influence of these two inducers. Notably, the oxidative phosphorylation process was most profoundly affected, with the expression of genes encoding NADH-coenzyme Q reductase (NADH-COQR1) and ATP synthase subunits being generally upregulated (Figure 4C). Further analysis uncovered differential regulation of the *ctaA* and *ctaB* genes, which encode subunits of cytochrome c oxidase complex IV, by H_2_S_n_ and H_2_O_2_. Specifically, under H_2_S_n_ induction, the expression of the *ctaA* gene was downregulated by 1.37- to 2.68-fold, whereas under H_2_O_2_ induction, it was downregulated by 0.43- to 1.99-fold. The *ctaB* gene exhibited a 1.00- to 2.75-fold decrease in expression under the H_2_S_n_ treatment, but no significant changes were observed under the H_2_O_2_ treatment. These findings suggest that H_2_S_n_ may more potently influence respiratory chain function by downregulating the expression of *ctaA* and *ctaB* genes.

The impact on the TCA cycle was relatively modest, with alterations observed in only two genes (Figure 4C). The *tpiA* gene, encoding triosephosphate isomerase (TPI), was upregulated by 0.31- to 1.81-fold under H_2_S_n_ induction and by 0.39- to 1.76-fold under H_2_O_2_ induction. TPI, a key enzyme in the glycolytic pathway, catalyzes the isomerization between dihydroxyacetone phosphate (DHAP) and glyceraldehyde-3-phosphate (G3P), playing a crucial role in maintaining glycolytic flux and energy conversion efficiency. The *icd* gene, encoding isocitrate dehydrogenase, was downregulated by 0.31- to 1.16-fold under H_2_S_n_ induction but showed no significant changes under H_2_O_2_ induction. Isocitrate dehydrogenase, which catalyzes the oxidative decarboxylation of isocitrate to α-ketoglutarate while reducing NAD^+^ or NADP^+^ to NADH or NADPH, plays a vital role in energy metabolism and immune responses.

H_2_S_n_ and H_2_O_2_ also influenced several enzymatic steps in the glycolysis pathway. For instance, the *gpmI* gene, encoding phosphoglycerate mutase (PGAM), exhibited an upregulation trend under both inducers (Figure 4C). Under H_2_S_n_ induction, the expression of *gpmI* was upregulated by 0.54- to 2.40-fold, whereas under H_2_O_2_ induction, it was upregulated by only 0.85- to 1.03-fold. This suggests that H_2_S_n_ may have a stronger activating effect on the glycolytic pathway. Additionally, key genes involved in the conversion of pyruvate to acetyl-CoA, including *pdhB, pdhC*, *pdhD*, and *nifA*, were regulated by H_2_S_n_ and H_2_O_2_. The *pdhB* and *pdhC* genes showed an upregulation trend, while *pdhD* and *nifA* exhibited a downregulation trend. This indicates that H_2_S_n_ and H_2_O_2_ may modulate the supply of substrates entering the TCA cycle by regulating the activity of the pyruvate dehydrogenase complex.

Overall, the results of this study demonstrate that H_2_S_n_ and H_2_O_2_ have the most pronounced impact on oxidative phosphorylation, with cells being more sensitive to H_2_S_n_, as evidenced by the greater magnitude of gene expression changes following H_2_S_n_ induction. These findings suggest that H_2_S_n_ may play a more significant role than H_2_O_2_ in regulating cellular energy metabolism. Collectively, this study provides new insights into the mechanisms by which H_2_S_n_ and H_2_O_2_ regulate cellular energy metabolism.

### 3.5. Effects of High Concentrations of H_2_S_n_ and H_2_O_2_ on Synechococcus sp. PCC7002 Growth and Their Tolerance Mechanisms

**Figure 5:**
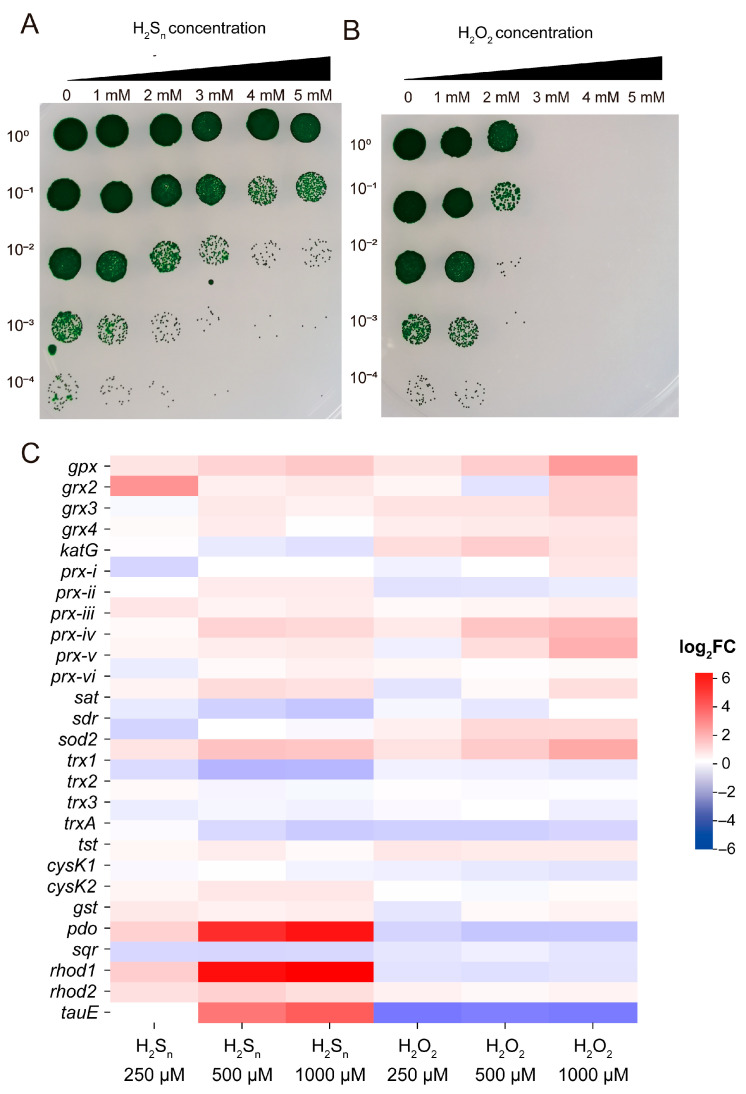
*Synechococcus* sp. PCC7002 showed different tolerance to H_2_S_n_ and H_2_O_2_. (**A**) The effect of H_2_S_n_ on the growth of *Synechococcus* sp. PCC7002; (**B**) The effect of H_2_O_2_ on the growth of *Synechococcus* sp. PCC7002; (**C**) Heat map of differentially expressed genes involved in H_2_Sn and H_2_O_2_ metabolism after 60 min treatment with 250, 500 or 1000 µM H_2_S_n_ (left) or H_2_O_2_ (right). Gene expression changes are shown as log_2_ FC relative to untreated controls; the color scale spans −6 (blue, strong repression) to +6 (red, strong induction). Core antioxidant genes (*sod2*, *katG*, *gpx*, *grx* and *prx* paralogs) were selectively upregulated by H_2_O_2_, whereas sulfur-handling genes (*sqr*, *cysK1/2*, *rhod1/2*) responded predominantly to H_2_S_n_.

High concentrations of H_2_S_n_ and H_2_O_2_ can induce oxidative stress in cells, thereby impairing normal growth. To evaluate *Synechococcus* sp. PCC7002’s tolerance to these compounds, we measured growth inhibition under different concentration treatments. The results demonstrated that *Synechococcus* sp. PCC7002 exhibited strong tolerance to H_2_S_n_: 1 mM of H_2_S_n_ only partially inhibited growth, and while the inhibitory effect gradually increased with higher concentrations (up to 5 mM), complete growth suppression was not achieved (Figure 5A). In contrast, *Synechococcus* sp. PCC7002 showed significantly higher sensitivity to H_2_O_2_, with 2 mM of H_2_O_2_ causing substantial growth inhibition and 3 mM leading to complete growth arrest (Figure 5B).

To elucidate the tolerance mechanisms, we analyzed expression changes in key metabolic enzymes related to H_2_S_n_ and H_2_O_2_ metabolism (Figure 5C). Under H_2_O_2_ stress, ROS metabolic genes (including *gpx, grx, prx, katG,* and *sod*) were significantly upregulated, while thioredoxin (*trx*) expression remained unchanged. In H_2_S_n_-treated groups, H_2_S_n_ metabolism-related genes (*pdo, rhod, tauE,* and *cysK2*) showed marked upregulation. Notably, *sqr* encoding sulfide quinone oxidoreductase (SQR), which catalyzes H_2_S oxidation to H_2_S_n_, was significantly downregulated, potentially reducing H_2_S_n_ accumulation. Interestingly, H_2_S_n_ also induced the upregulation of certain ROS metabolic genes (*gpx, grx, prx,* and *sod*). In conclusion, H_2_S_n_ not only specifically activates the RSS metabolic pathway but also partially induces adaptive responses in the ROS detoxification system. This dual regulatory mechanism likely underlies *Synechococcus* sp. PCC7002’s superior H_2_S_n_ tolerance. These findings provide important molecular insights into *Synechococcus* sp. PCC7002’s survival strategies under high H_2_S_n_ or H_2_O_2_ conditions.

## 4. Discussion

In this study, we provide direct experimental evidence that *Synechococcus* sp. PCC 7002 can perform anoxygenic photosynthesis via the SQR-mediated oxidation of H_2_S by combining targeted gene deletion (Δ*sqr*) with the DCMU-mediated inhibition of oxygenic photosynthesis (Figure 1). Subsequent transcriptomic analyses revealed that H_2_O_2_ and H_2_S_n_ act as distinct signaling molecules to differentially modulate the expression of key genes involved in photosynthesis, TCA cycle, glycolysis and oxidative phosphorylation (Figure 3 and Figure 4). Notably, although H_2_O_2_ and H_2_S_n_ display partially overlapping regulatory functions, cells exhibit a markedly higher sensitivity toward RSS. Of particular interest, *Synechococcus* sp. PCC7002 demonstrates significantly greater tolerance to H_2_S_n_ than to H_2_O_2_, a phenotype likely attributable to the dual regulatory role played by H_2_S_n_, simultaneously activating sulfur metabolism pathways and inducing antioxidant defense systems (Figure 5).

This study provides the first direct experimental confirmation of anoxygenic photosynthetic capacity within the genus *Synechococcus*. Although earlier studies have postulated that cyanobacteria might perform anoxygenic photosynthesis under anoxic, sulfide-rich conditions [7,42,43], definitive evidence remains elusive. We previously demonstrated that *Synechococcus* sp. PCC7002 encodes a functional SQR capable of oxidizing H_2_S to polysulfide [23]. Building upon this finding, we now establish that *Synechococcus* sp. PCC7002 can indeed utilize SQR to oxidize H_2_S for anoxygenic photosynthesis (Figure 1). The ability of cyanobacteria to switch between oxygenic and anoxygenic photosynthesis has profound ecological implications. In the low-oxygen environment of the early days of the Earth, anoxygenic photosynthesis conferred a selective advantage, setting the stage for the subsequent evolution of oxygenic photosynthesis [44,45]. The eventual transition to oxygenic photosynthesis, involving the splitting of water and the release of O_2_, transformed Earth’s atmosphere from reducing to oxidizing, thereby enabling the radiation of aerobic life [46]. Contemporary Cyanobacteria retain the capacity for anoxygenic photosynthesis, enabling them to thrive in specialized niches characterized by anoxia and elevated sulfide levels, thereby expanding their ecological breadth.

H_2_S_n_ and H_2_O_2_ participate as signaling molecules in the regulation of numerous metabolic processes, yet their mechanisms of action appear to be different. For example, the *psbA* and *petC* genes displayed opposing expression patterns following H_2_S_n_ or H_2_O_2_ treatment. *psbA* encodes the D1 protein of photosystem II [47,48], whereas *petC* encodes a subunit of the cytochrome b6f complex [49]. Both genes were upregulated by H_2_S_n_ but downregulated by H_2_O_2_. This antagonistic transcriptional response suggests that the two molecules modulate photosynthesis via distinct pathways. H_2_S_n_ may enhance photosynthetic efficiency and cellular antioxidant capacity by activating antioxidant defenses and upregulating photosynthesis-related genes [50,51,52], thereby sustaining photosynthetic activity under elevated H_2_S_n_. Conversely, H_2_O_2_, a potent oxidant, may trigger oxidative-stress responses and repress photosynthesis-associated genes to minimize ROS production during photosynthesis [53,54], thus protecting cells from oxidative damage. These opposing patterns illuminate the complex regulatory networks underlying photosynthetic modulation, providing a molecular framework for understanding the fine-tuned control of photosynthesis.

Our investigation of the effects of H_2_S_n_ and H_2_O_2_ on the TCA cycle, glycolysis and oxidative phosphorylation revealed that both molecules predominantly influence oxidative phosphorylation, specifically upregulating genes encoding NADH–CoQ reductase (NADH-COQR1) and ATP synthase [55,56,57]. NADH–CoQ reductase (complex I) catalyzes electron transfer from NADH to coenzyme Q while translocating protons across the membrane to generate the proton-motive force required for ATP synthesis [58]. ATP synthase subsequently utilizes this proton gradient to synthesize ATP. Our transcriptomic data indicate that H_2_S_n_ and H_2_O_2_ upregulate the expression of NADH-COQR1 and ATP synthase genes (*ndh* series) [59,60]. This transcriptional activation increases the abundance of these proteins, thereby enhancing electron transport chain activity and elevating ATP production to meet the cellular energy demands. Additionally, H_2_S_n_ and H_2_O_2_ modulate the expression of the genes involved in glycolysis and the TCA cycle, conferring greater metabolic flexibility and facilitating adaptation to environmental fluctuations. These findings highlight the pivotal roles played by H_2_S_n_ and H_2_O_2_ in the regulation of cellular energy metabolism and provide molecular insights into the adaptive mechanisms employed under oxidative stress.

A particularly noteworthy observation of this study is that *Synechococcus* sp. PCC7002 exhibits substantially higher tolerance to H_2_S_n_ than to H_2_O_2_. Transcriptomic analyses revealed that H_2_S_n_ not only induces sulfur metabolism genes but also upregulates ROS-detoxifying enzymes, which may account for the enhanced tolerance. Numerous studies have shown that the enzymes traditionally associated with ROS metabolism are also capable of processing RSS [61,62,63]. From an evolutionary perspective, the sulfide-rich environment of the early Earth may have driven the primordial emergence of RSS-processing systems, whereas ROS-detoxifying mechanisms evolved later in response to the Great Oxidation Event (GOE) [64,65]. The conservation of such metabolic systems thus offers molecular evidence of the transition of life from anoxic to oxic conditions.

## 5. Conclusions

In summary, this study not only experimentally confirms *Synechococcus* sp. PCC 7002’s capacity to perform anoxygenic photosynthesis but also elucidates how the photosynthetic intermediates H_2_S_n_ and H_2_O_2_ differentially regulate photosynthesis, the TCA cycle, and related metabolic pathways. Our findings reveal both the shared and distinct regulatory roles played by H_2_S_n_ and H_2_O_2_. Moreover, we dissect the mechanistic basis for *Synechococcus* sp. PCC7002’s superior tolerance to high H_2_S_n_ concentrations, demonstrating that elevated H_2_S_n_ simultaneously induces ROS and RSS metabolizing enzymes. Collectively, our genome-wide analysis provides a comprehensive understanding of the metabolic regulatory networks underlying distinct photosynthetic modes in *Synechococcus*, establishes a foundation for investigating the adaptive transition of cyanobacteria from anoxic to oxic environments, and offers mechanistic insights into the resilience of Cyanobacteria under fluctuating environmental conditions.

## Data Availability

Raw sequencing reads were deposited in the NCBI Sequence Read Archive (SRA) under the project number PRJNA1289363.

## Supplementary Materials

The following supporting information can be downloaded at https://www.mdpi.com/article/10.3390/antiox14091122/s1. Figure S1: *Synechococcus* sp. PCC 7002 used H2S as electron donor to perform anoxygenic photosynthesis. Figure S2: Per-base quality scores for the full-length reads. Figure S3: Principal coordinates analysis (PCoA) of gene-expression profiles (log-transformed FPKM values) based on Bray–Curtis dissimilarity under the indicated H_2_O_2_ and H_2_S_n_ treatments. Figure S4: The transcriptional response of *Synechococcus* sp. PCC7002 to 250 µM and 500 µM H_2_S_n_/H_2_O_2_. Figure S5: KEGG pathway enrichment analyses of DEGs following 250 µM and 500 µM H_2_S_n_ (A,B)/H_2_O_2_ (C,D) exposure.
