# Supplementary material for: Synechococcus sp. PCC 7002 Performs Anoxygenic Photosynthesis and Deploys Divergent Strategies to Cope with H2Sn and H2O2
Source: Antioxidants (Basel). 2025 Sep 16;14(9):1122. doi: 10.3390/antiox14091122 (PMC12466478; doi:10.3390/antiox14091122)
Supplement: Supplementary file 1 [file antioxidants-14-01122-s001.zip › antioxidants-3857485-supplementary.pdf]

Synechococcus sp. PCC 7002 performs anoxygenic photosynthesis and deploys divergent strategies to cope with H_2Sn and H_2O_2

Yafei Wang [1,2†], Yue Meng [1,2†], Hongwei Ren [3], Ranran Huang [2], Jihua Liu [2], Daixi Liu [1,*]

[1] State Key Laboratory of Discovery and Utilization of Functional Components in Traditional Chinese Medicine, Key Laboratory of Chemical Biology (Ministry of Education), Shandong Basic Science Research Center (Pharmacy), School of Pharmaceutical Sciences, Cheeloo College of Medicine, Shandong University, Jinan 250012, China
[2] Institute of Marine Science and Technology, Shandong University, Qingdao 266237, China
[3] Key Laboratory of Land and Sea Ecological Governance and Systematic Regulation, Ministry of Ecology and Environment, Shandong Academy for Environmental Planning, Jinan 250101, China
* Correspondence:
Daixi Liu: liudaixi@sdu.edu.cn

†These authors contributed equally to this work and shared first authorship

Supplementary materials

Figure S1. ***Synechococcus*** **sp. PCC 7002 used H_2S as electron donor to perform anoxygenic photosynthesis.**

Figure S2. Per-base quality scores for the full-length reads.

Figure S3. Principal coordinates analysis (PCoA) of gene-expression profiles (log-transformed FPKM values) based on Bray-Curtis dissimilarity under the indicated H_2O_2 and H_2S_n treatments.

Figure S4. The transcriptional response of ***Synechococcus*** **sp. PCC7002 to 250 μM and 500 μM H_2S_n/H_2O_2.**

Figure S5. KEGG pathway enrichment analyses of DEGs following 250 μM and 500 μM H_2S_n (A, B)/H_2O_2 (C, D) exposure.

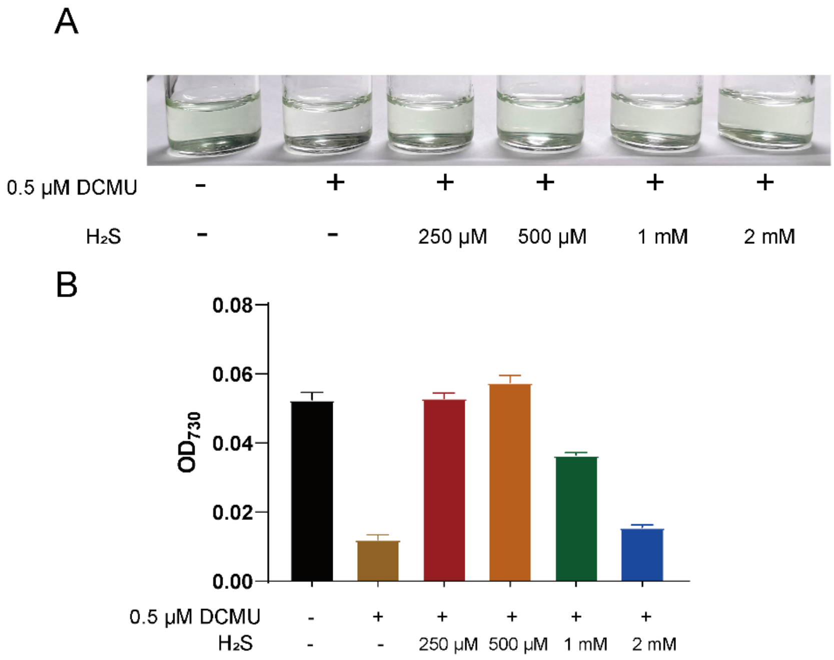

Figure S1. Synechococcus sp. PCC 7002 used H2S as electron donor to perform anoxygenic photosynthesis. 0.5 μM DCMU and multiple concentrations of sulfide were added to the Synechococcus sp. PCC 7002 culture, then the images (A) and densities (B) of Synechococcus sp. PCC 7002 were tested after culturing for 10 days under anaerobic condition.

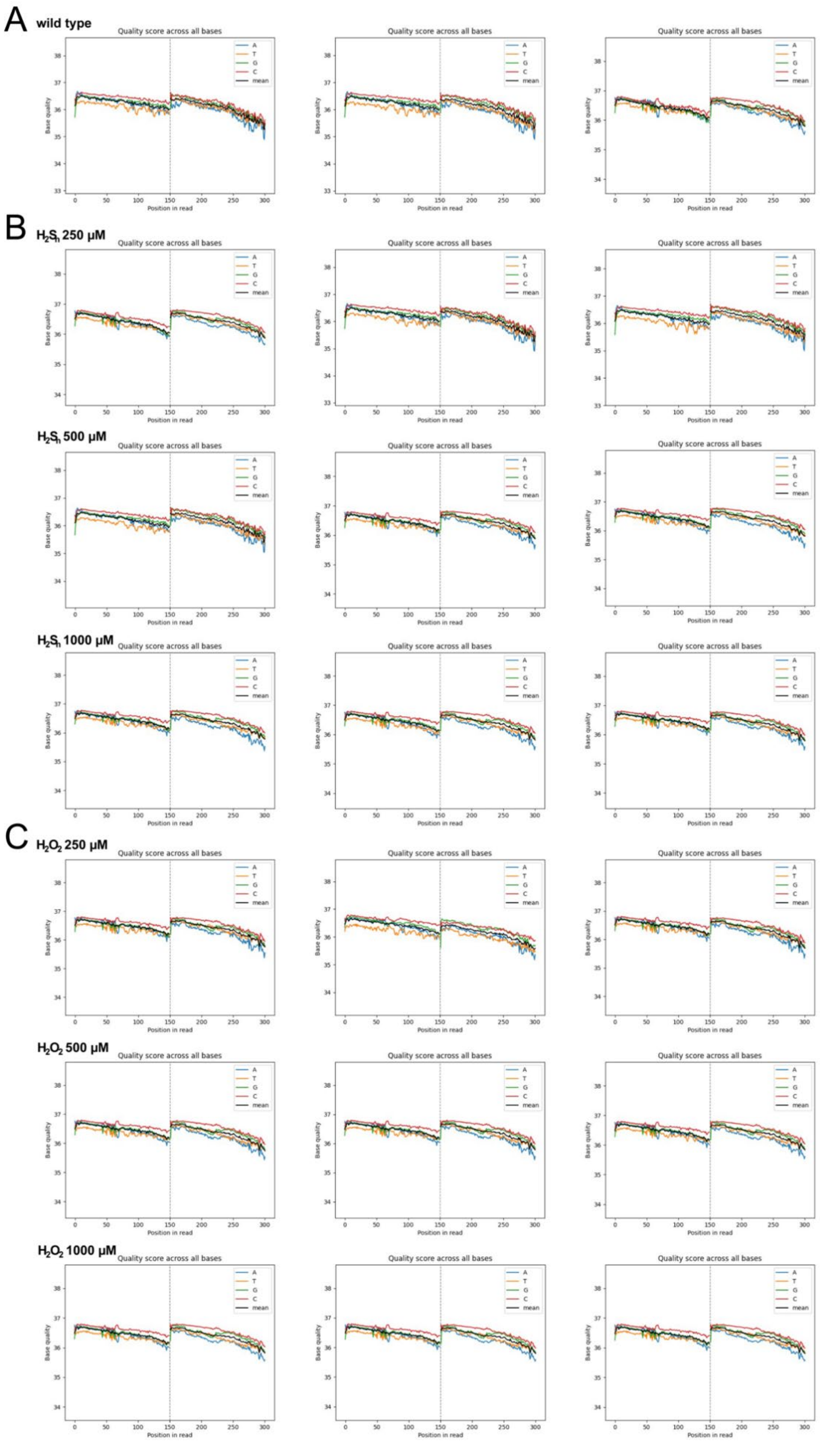

Figure S2. Per-base quality scores for the full-length reads. Each treatment group (wild type; 250, 500, 1000 µM H_2S; 250, 500, 1000 µM H_2O_2) is represented by three biological replicates, shown as individual lines.

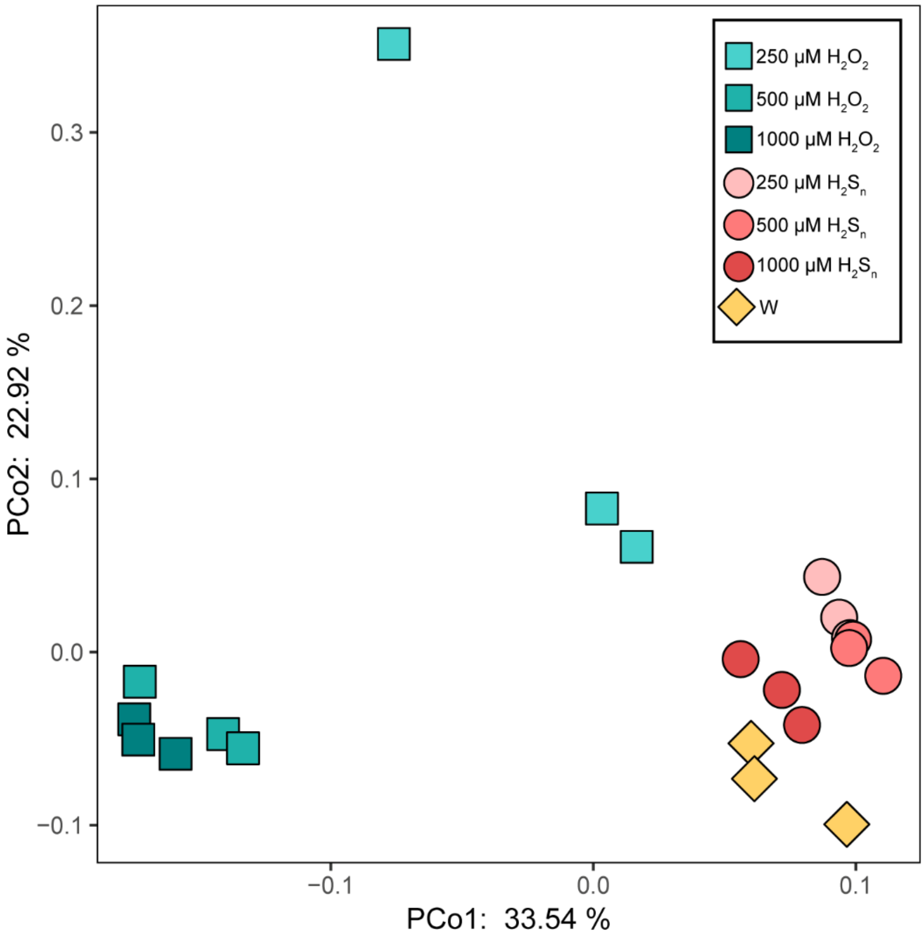

Figure S3. Principal coordinates analysis (PCoA) of gene-expression profiles (log-transformed FPKM values) based on Bray-Curtis dissimilarity under the indicated H_2O_2 and H_2S_n treatments.

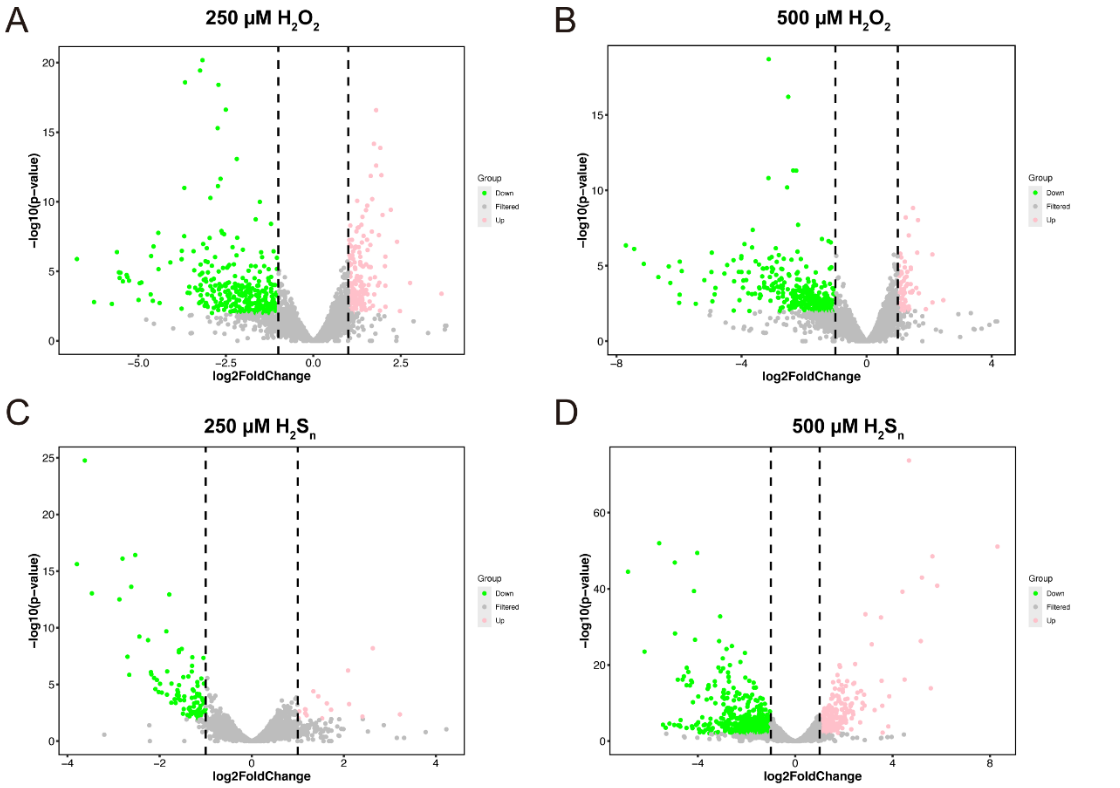

Figure S4. The transcriptional response of *Synechococcus* sp. PCC7002 to 250 μM and 500 μM H_2S_n/H_2O_2. (A, B) Volcano plots displaying differentially expressed genes (DEGs) after 250 μM (A) and 500μM H_2O_2 (B) treatment for 60 min. **(C, D)** Volcano plots displaying differentially expressed genes (DEGs) after 250 μM (C) and 500μM H_2S_n (D) treatment for 60 min. Horizontal dashed lines denote the adjusted p-value threshold (padj < 0.05); vertical dashed lines mark $|\log_2 FC| \geq 1$.

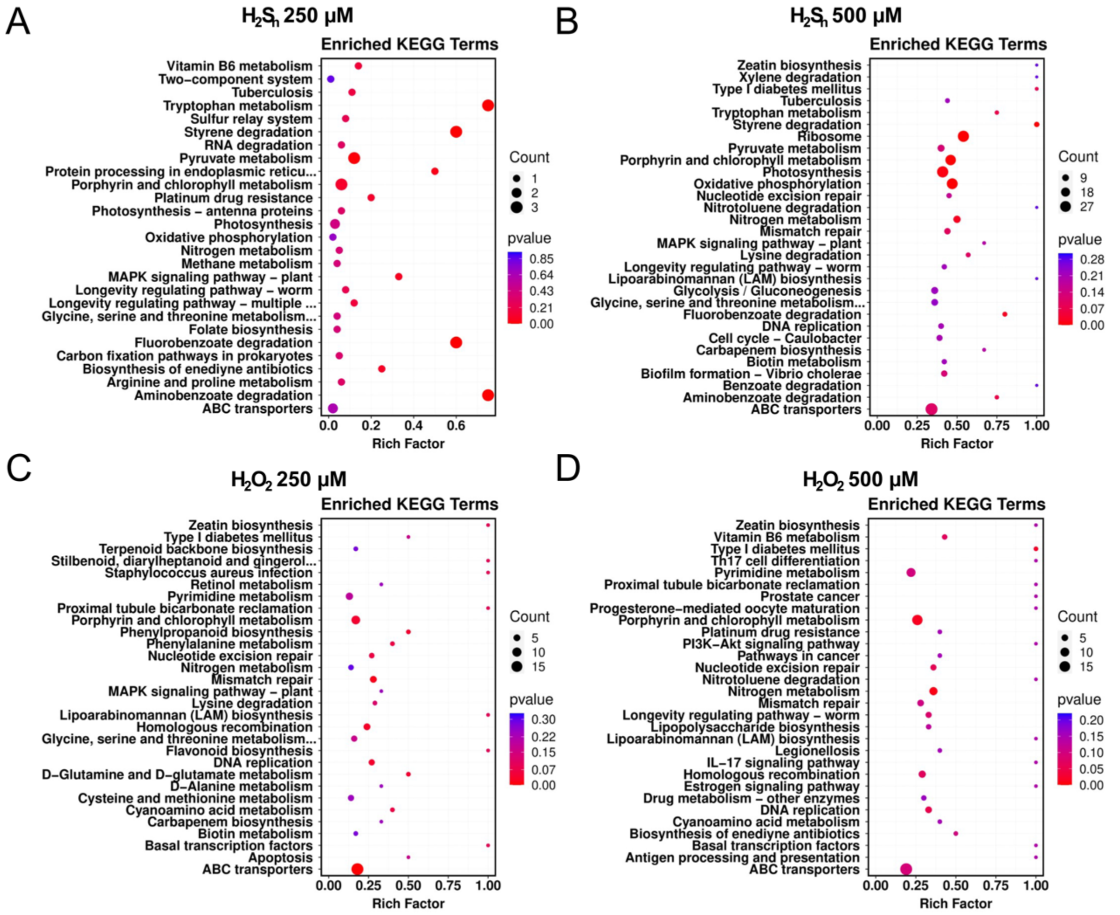

Figure S5. KEGG pathway enrichment analyses of DEGs following 250 μM and 500 μM H_2S_n (A, B)/H_2O_2 (C, D) exposure. Bubble size indicates the number of enriched genes; color intensity represents -$\log_{10}$ (pvalue); and the x-axis (RichFactor) reflects the proportion of DEGs relative to all genes in each pathway.
